# The Diagnostic and Prognostic Potential of the B-Cell Repertoire in Membranous Nephropathy

**DOI:** 10.3389/fimmu.2021.635326

**Published:** 2021-05-27

**Authors:** Zuhui Su, Yabin Jin, Yu Zhang, Zhanwen Guan, Huishi Li, Xiangping Chen, Chao Xie, Chuling Zhang, Xiaofen Liu, Peixian Li, Peiyi Ye, Lifang Zhang, Yaozhong Kong, Wei Luo

**Affiliations:** ^1^ Clinical Research Institute, The First People’s Hospital of Foshan, Foshan, China; ^2^ Nephrology Department, The First People’s Hospital of Foshan, Foshan, China

**Keywords:** B-cell receptor repertoire, membranous nephropathy, high-throughput sequencing, immunoglobulin heavy chain, biomarkers

## Abstract

Membranous nephropathy (MN), an autoimmune glomerular disease, is one of the most common causes of nephrotic syndrome in adults. In current clinical practice, the diagnosis is dependent on renal tissue biopsy. A new method for diagnosis and prognosis surveillance is urgently needed for patients. In the present study, we recruited 66 MN patients before any treatment and 11 healthy control (HC) and analyzed multiple aspects of the immunoglobulin heavy chain (IGH) repertoire of these samples using high-throughput sequencing. We found that the abnormalities of CDR-H3 length, hydrophobicity, somatic hypermutation (SHM), and germ line index were progressively more prominent in patients with MN, and the frequency of *IGHV3-66* in post-therapy patients was significantly lower than that in pre-therapy patients. Moreover, we found that the *IGHV3-38* gene was significantly related to PLA2R, which is the most commonly used biomarker. The most important discovery was that several *IGHV*, *IGHD* transcripts, CDR-H3 length, and SHM rate in pre-therapy patients had the potential to predict the therapeutic effect. Our study further demonstrated that the IGH repertoire could be a potential biomarker for prognosis prediction of MN. The landscape of circulating B-lymphocyte repertoires sheds new light on the detection and surveillance of MN.

## Introduction

Membranous nephropathy (MN) is the most common cause of idiopathic nephrotic syndrome in non-diabetic adults worldwide, representing between 20 and 37% in most series and rising to as high as 40% in adults over 60 (1, 2). MN is characterized by thickening of the glomerular basement membrane (GBM) and rigid capillaries (3). It is widely accepted that MN is an autoimmune reaction to inherent podocyte antigens. The antigenic targets of these antibodies are most often PLA2R and THSD7A (4, 5).

Clinically, the diagnosis of MN depends on the histopathologic features of renal biopsy. However, renal biopsy is an invasive procedure, and it could increase the potential for kidney infection. Moreover, during pre- and post-treatments, repeating kidney biopsies to monitor disease progression is inconvenient. Patients badly need a dependable and minimally invasive biomarker to diagnose MN, assess disease stage, and evaluate treatment effect. Thanks to recent advances, we now know that some novel biomarkers have been developed, and the most commonly used biomarker is APLA2R (6–8). APLA2R has shown some potential in MN, but there are still some false positives and false negatives. As a result, more novel biomarkers are needed.

In recent years, arguments about MN have led to the conclusion that B cells play a key role in the disease, and the treatment targeting the B lymphocyte could be more significant. Rituximab (RTX) has been a novel anti-CD20 monoclonal antibody in patients with nephrotic syndrome due to MN (9). After RTX, the use of belimumab is a new step in B-lymphocyte-targeting therapy (10). Belson et al. (11) assessed the effect of belimumab on proteinuria and PLA2R serology in patients with PLA2R-related MN. These treatments targeting the B lymphocyte indicated that the pathogenesis of MN was strongly associated with B-lymphocyte repertoire.

At present, many studies using B-cell receptor repertoire high-throughput sequencing (BCR-HTS) have characterized the B-cell repertoires and demonstrated their diagnostic, therapeutic, and prognostic significance in different immune-related diseases, including autoimmune diseases (12), immunoglobulin A nephropathy (13), and gastric cancer (14). The B-lymphocyte repertoire could reflect the B-cell immune condition in MN, and sequencing the B-cell receptors to investigate the diversity of B lymphocytes provided a new path to understand the pathogenesis of the disease.

Numerous studies have focused mostly on the immune status of immunoglobulin A nephropathy rather than on MN. Chen Huang et al. (13) presented a comprehensive landscape of T-cell receptor beta chain (TCR*β*) and IGH in immunoglobulin A nephropathy patients. Dapeng Chen et al. (15) analyzed the CDR-H3 clones of immunoglobulin A nephropathy patients and Minglin Ou et al. (16) also reported B- and T-cell repertoires CDR3 sequences in immunoglobulin A nephropathy patients. Compared to it, there are few studies about IGH genes in MN.

In this study, we analyzed the IGH repertoire of healthy control (HC) and pre- and post-therapy MN patients by BCR-HTS. We found that six *IGHC*, one *IGHD*, and 24 *IGHV* genes were significantly different between MN patients and HC. For the *IGHV* genes, most of them belong to the *IGHV3* and *IGHV4* families. The analysis of the CDR-H3 length, hydrophobicity, and somatic hypermutation (SHM) also revealed significant difference between MN patients and HC. Moreover, we found that the frequency of *IGHV3-66* in post-therapy patients was significantly lower than that in pre-therapy patients, and *IGHV3-38* genes were significantly related to PLA2R, which is the commonly used biomarker. Importantly, we identified 12 *IGHV* genes, and two *IGHD* genes were significantly different between complete remission (CR) and non-CR patients. Moreover, the CDR-H3 length and SHM rate in the IgM subtype were observed to be higher in the CR group. All the results demonstrated that the IGH repertoire has the potential to be a novel biomarker for prognosis prediction in MN.

## Methods

### Samples

This study was approved by the Ethics Committee of the Affiliated Foshan Hospital of Sun Yat-Sen University. From 2017 to 2018, we recruited 66 patients with MN before any treatment. Clinical characteristics of the MN patients are shown in **Tables 1** and **S1**. The disease diagnosis criterion of MN was established by renal biopsy. The kidney tissue biopsies of 66 patients were examined by light microscopy, electron microscopy, and immunofluorescence. Peripheral blood samples were collected before treatment for the 66 patients. Among them, the peripheral blood samples of 13 patients were collected at 6 months after treatment. Assessment of disease state indicators included 24 h urine protein, serum albumin, PLA2R status, uric acid, and serum creatinine, which were collected in renal biopsy and the follow-up at 6 months after therapy. According to Kidney Disease: Improving Global Outcomes (KDIGO), we evaluated the prognostic condition of the MN patients (17, 18). Without a history of cancer, autoimmune disorder, or surgery, 11 healthy volunteers were recruited as HC, and peripheral blood samples were drawn from each person. Peripheral blood mononuclear cells (PBMCs) were isolated immediately and lysed with TRIzolR reagent (Life, US), then frozen at −80°C.

**Table 1:** Summary of clinical characteristics of the patients and healthy control.

Variable	MN patients	Healthy control	P-value
**Gender (female/male)**	24/42	2/9	0.316
**Age (years)**	51(16–69)	48(24–61)	0.325
**24 h UP (g)**	4.15(0.32–15.9)	/	/
**Serum albumin (g/L)**	26.85(15.7–42.1)	/	/
**PLA2R (Ru/ml) **	76.39(0.6–1500)	/	/
**Serum creatinine (µmol/L)**	76.5(38–165)	/	/
**serum uric acid (µmol/L)**	413(181–848)	/	/

### High-Throughput Sequencing of IGH Repertoire

Following the manufacturer’s instructions, we extracted RNA from PBMC lysates using a total RNA Kit (OMEGA Bio-tek, US). For each sample, we used the SMARTer PCR cDNA synthesis kit (Clontech, US) to reverse transcribe 1 µg total RNA into cDNA. To amplify a fully sequenced IGH strand fragment, the 5′RACE-ready cDNA was used as a template for a 5′RACE-PCR with forward universal primer and reverse mix primers specific to the IGH constant region C*α* (*IGHA*), C*μ* (*IGHM*), C*γ* (*IGHG*), C*δ* (*IGHD*), and C*ϵ* (*IGHE*) (The primer list is shown in **Table S3**). The PCR conditions were as follows: 3 min denaturation at 94°C was followed by 40 cycles of 15 s at 94°C, 30 s at 58°C, and 45 s at 72°C, plus a final extension for 10 min at 72°C. Next, we used the gel extraction kit (QIAGEN, German) to purify the products by 2% agarose gel electrophoresis. Illumina HiSeq sequence adaptors were ligated to construct sequencing libraries, which were then sequenced on an Illumina HiSeq Xten platform. Data from the IGH repertoire library sequencing can be provided on request.

### Bioinformatics Analysis of the IGH Repertoire HTS Data

The sequencing data was stored in a FASTQ format. After filtering low-quality sequences, the high-quality sequences were aligned with the BCR reference sequences by BLAST (-stepSize = 5 –minIdentity = 0 –minScore = 0) (19). The reference sequences were downloaded from the IMGT/GENE database (20). If V, J, and C genes in a given sequence were all identified, we further translated them into an amino acid (aa) sequence. The aa sequences without a terminator were selected as the productive BCR sequences. At the V–D–J junctions, the sequences that started with cysteine and ended with the tryptophan were defined as CDR-H3. The source code of our own BCR sequence bioinformatical analysis tool is available on request.

### Indices in This Study

We selected the Shannon index and Simpson index to estimate the diversity in the BCR repertoire. Both of them take into consideration the two components that constitute the concept of diversity, the richness of a population and its homogeneity. The richness of a population is defined by its total number of species, and homogeneity measures the distribution of the species (21–23). The formulas of the Shannon index and Simpson index are as follows:

$$\begin{array}{c} \text{Shannon index}\;=-\sum_{i}\left(\frac{n_{i}}{N}\right){\text{log}}_{2}\left(\frac{n_{i}}{N}\right);\\ \text{Simpson index}\;=1/\sum_{i}{\left(n_{i}/N\right)}^{2}; \end{array}$$

where *i* is an index that is chosen between 1 and the number of species *s*; *n_i_* is the number of sequencing reads in species *i*; and N is the total number of reads.

Kyte–Doolittle index of hydrophobicity in CDR-H3 was calculated based on the normalized Kyte–Doolittle scale which assigned one value to each amino acid (24). SHM introduces additional diversity in the IGH repertoire of mature B cells and allows selection of high-affinity antibodies. SHM rate was calculated by dividing the number of point mutations by the total number of nucleotides in the V gene of our sequences (25). The germline index (GI) can be used to estimate the abundance of palindromic and “N” nucleotides and was calculated by dividing the number of nucleotides in the CDR3 that were encoded by V, D, and J genes by the total number of nucleotides contained in the CDR3, generating a value between 0 and 1 (26).

### Statistical Analysis

Comparisons between groups were conducted using the Mann–Whitney U test or the Wilcoxon signed rank test if appropriate, and P <0.05 was considered statistically significant. The Spearman correlation coefficient (r) was used to measure the linear correlation between pairs of variables. These analyses were performed using GraphPad Prism software (version 5.1), SPSS (version 20.0), and R software (version 3.4.1; http://www.Rproject.org).

## Results

### Clinical Characteristics of the Patients

The demographic and clinical characteristics of the patients are summarized in **Tables 1** and **S1**. With regard to the pre-therapy MN patients, 42 of them were male, and 24 of them were female. The average age of the patients was 51 years old. There we no significant differences in gender and age between the MN patients and HC. The average 24 h proteinuria measurement was 4.15 g for MN patients and varied largely among patients (0.32 g/24 h–15.9 g/24 h). Serum creatinine, serum albumin, uric acid, and PLA2R also varied extensively among MN patients, with an average level of 76.5 µmol/L, 26.8 g/L, 413 µmol/L, and 76.39 Ru/ml, respectively.

### Profiling of the IGH Sequencing Data

**Figure 1:**
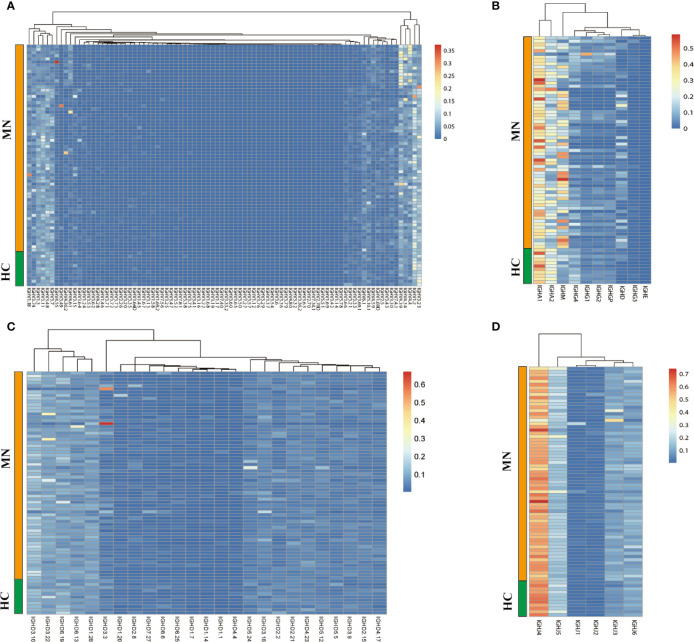
The heat maps of IGH gene usage frequencies in BCR repertoire from the sample of the MN patients and HC. **(A)**: *IGHV*; **(B)**: *IGHC*; **(C)**: *IGHD*; **(D)**: *IGHJ*.

A total of 119,300,197 productive aa sequences were obtained from 92 blood samples of the patients and HC, with an average of 1,296,741 productive sequences generated per sample. The average number of productive unique sequences per sample was 270,904. Eighty-six distinct *IGHV*, 25 distinct *IGHD*, and 10 distinct *IGHC* segments were identified, and the usage frequencies of these segments were analyzed in each sample, which are shown in **Figure 1** and **Table S2**.

### Increased *IGHM*, *IGHD*, and *IGHE* Frequencies and Decreased *IGHA* and *IGHG4* Frequencies in PBMCs for MN Patients

**Figure 2:**
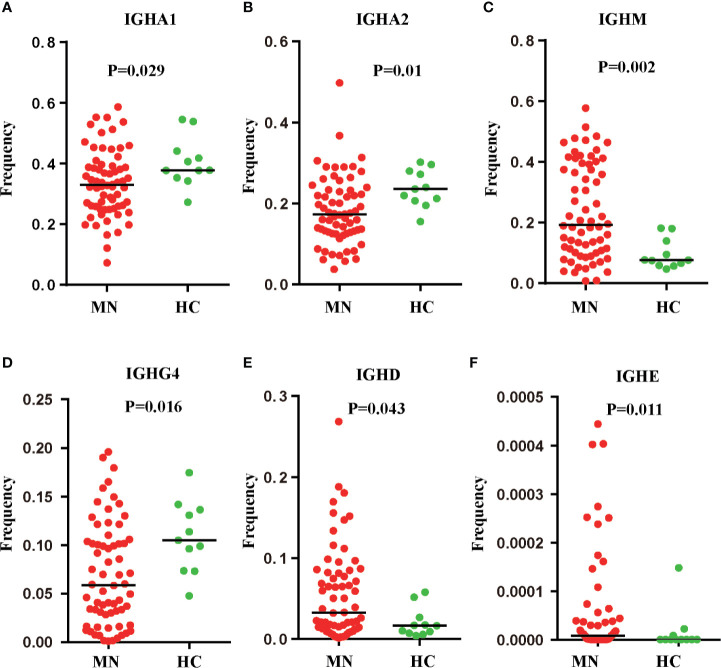
Six IGH transcripts were significantly different between MN patients and HC. *IGHA1* **(A)**, *IGHA2* **(B)**, *IGHM* **(C)**, *IGHG4* **(D)**, *IGHD* **(E)**, *IGHE* **(F)** frequency distribution in PBMC of MN patients (n = 66) and HC (n = 11) with line at the median.

We compared the *IGHC* frequency between MN patients and HC. The frequency was delineated in heat maps (**Figure 1A**). There are six transcripts (*IGHA1, IGHA2, IGHM, IGHG4, IGHD, IGHE*) in **Figure 1** that are observably different between MN patients and HC. *IGHA* is composed of two subclasses: *IGHA1* and *IGHA2*. A more detailed description is presented in **Figure 2**. We found that the frequency of *IGHM*, *IGHD*, and *IGHE* in PBMCs was significantly higher than those in MN patients. In contrast, the frequencies of *IGHA* and *IGHG4* were significantly lower in MN patients than in the HC. It showed that, compared with HC, the proportion of IG in the BCR repertoire of MN patients has changed. This result is consistent with previously reported results (27).

### The Different Usage Patterns of *IGHV* and *IGHD* Genes in MN Patients

**Figure 3:**
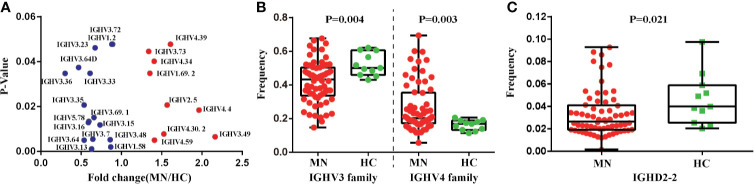
The *IGHV* and *IGHD* gene usage between MN patients and the HC. **(A)** Twenty-four *IGHV* genes which are significantly different between MN patients and the HC (without correction for multiple comparison). **(B)** The frequencies of *IGHV3* family and *IGHV4* family have significant differences in MN patients and the HC. **(C)** The frequency of *IGHD2-2* has significant differences in MN patients and the HC (p = 0.021).

We also compared the *IGHV*, *IGHD*, and *IGHJ* gene usage between MN patients and HC. There were 24 *IGHV* genes that were significantly different between the two groups (*P* < 0.05; **Figure 3A**; *IGHV3-72*, *IGHV1-2*, *IGHV3-23*, *IGHV3-64D*, *IGHV3-36*, *IGHV3-33*, *IGHV3-35*, *IGHV3-69-1*, *IGHV5-78*, *IGHV3-16*, *IGHV3-15*, *IGHV3-7*, *IGHV3-48*, *IGHV3-64*, *IGHV3-13*, *IGHV1-58*, *IGHV4-39*, *IGHV3-73*, *IGHV4-34*, *IGHV1-69-2*, *IGHV2-5*, *IGHV4-4*, *IGHV4-30-2*, *IGHV4-59*, *IGHV3-49*). We investigated the frequency of the *IGHV3* and *IGHV4* families and found that the frequency of the *IGHV3* family was lower in MN patients, but the frequency of the *IGHV4* family was higher than that in HC, with significant difference (**Figure 3B**). Moreover, we found the frequency of the *IGHD2-2* was significantly lower in MN patients (*P* = 0.021; **Figure 3C**). These results demonstrated that usage patterns of *IGHV* and *IGHD* genes were skewed in patients, and the B-cell immune status in MN patients had been altered.

### The CDR-H3 Length, Hydrophobicity, SHM Rate and GI of MN Patients Showed Significant Difference Compared With HC

**Figure 4:**
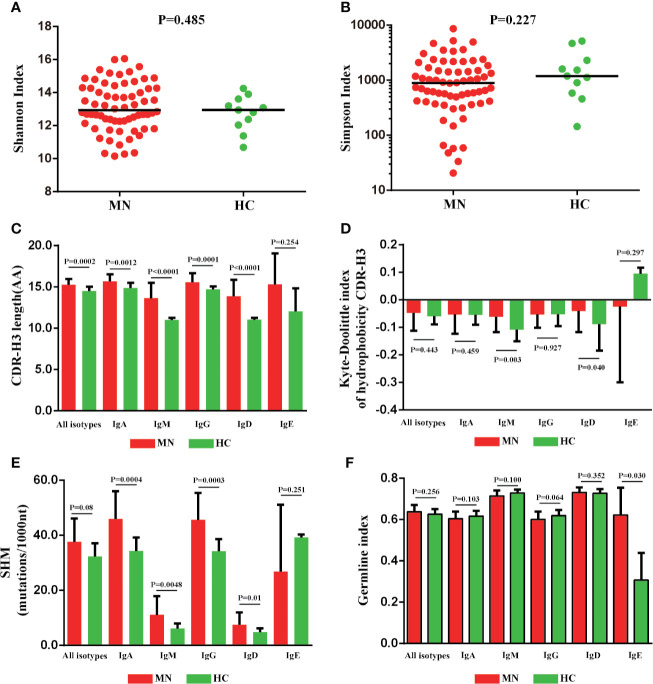
The CDR-H3 length, hydrophobicity somatic hypermutation and GI of MN patients showed significant differences compared with HC. The Shannon index **(A)**, Simpson index **(B)**, CDR-H3 length distribution **(C)**, the Kyte–Doolittle index of hydrophobicity **(D)**, the rate of SHM **(E)**, and the germline index **(F)** of IGH repertoire in MN patients and HC.

We compared the CDR-H3 length, hydrophobicity, SHM, and GI between MN patients and HC (**Figures 4A–F**); the CDR-H3 length distribution of *IGHA*, *IGHM*, *IGHG*, and *IGHD* transcripts was observed to be more prominent in patients with MN (*P* < 0.05; **Figure 4C**), and we found abnormalities of the hydrophobicity profile of the IGHM and IGHD in MN patients, as measured by the normalized Kyte–Doolittle index (*P* < 0.05; **Figure 4D**). In particular, MN patients showed a higher rate of SHM in *IGHA*, *IGHM*, *IGHG*, and *IGHD* transcripts compared with HC (*P* < 0.05; **Figure 4E**). Overall, these results showed the CDR-H3 length, hydrophobicity, and SHM had a significant difference between the patients with MN and HC. It indicated that CDR-H3 length, hydrophobicity, and SHM of the peripheral BCR repertoire were influenced significantly by MN.

### The Usage Patterns of *IGHV* Genes, *IGHD* Genes, CDR-H3 Length, and SHM Are Related to Therapy and Therapeutic Effect

**Figure 5:**
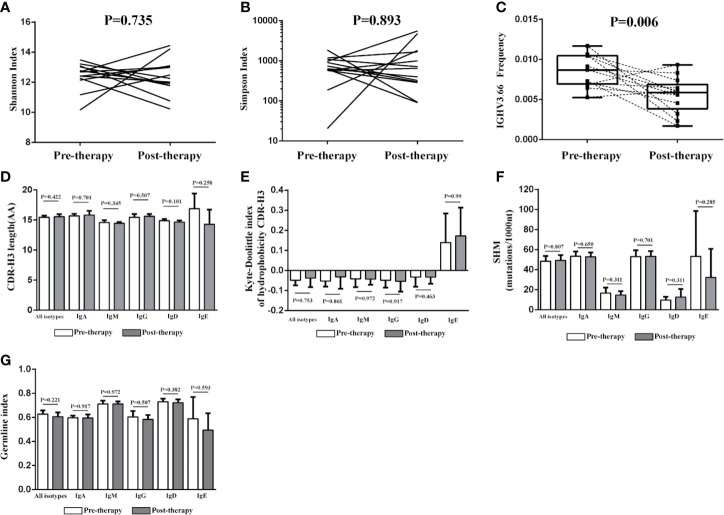
Change of the IGH repertoire between the pre-therapy and post-therapy patients. The Shannon index **(A)**, Simpson index **(B)**, CDR-H3 length distribution **(D)**, the Kyte–Doolittle index of hydrophobicity **(E)**, the rate of SHM **(F)** and the germline index **(G)** of IGH repertoire have no significant differences between pre-therapy and post-therapy, but the frequency of *IGHV3-66* was significantly lower in the samples of post-therapy patients (n = 13) than paired pre-therapy samples (n = 13) **(C)**.

When comparing the BCR repertoire between the pre- and post-therapy patients, no significant difference was observed for the diversity index, CDR-H3 length, SHM, hydrophobicity, GI, *IGHD* genes, *IGHJ* genes, and *IGHC* genes. However, we found that the frequency of *IGHV3-66* was significantly lower in post-therapy patients than that in pre-therapy patients (**Figure 5**).

**Figure 6:**
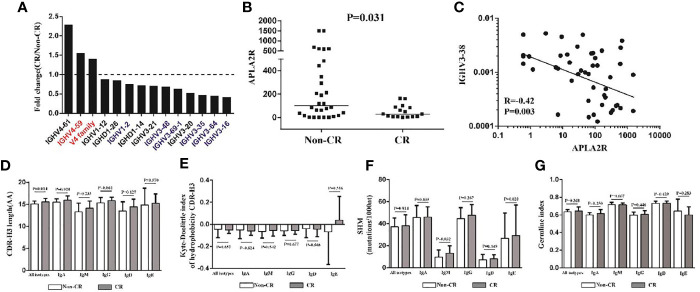
The *IGHV* genes, CDR-H3 length, SHM rate, and APLA2R between the CR and non-CR patients. **(A)** Three *IGHV* genes were significantly increased in the CR group (n = 22), two of them in MN patients are significantly higher than in HC, which is represented by red. Nine *IGHV* genes and two *IGHD* genes that were significantly lower than non-CR (n = 43), and six of them in MN patients are significantly higher than in HC, which is represented by blue. **(B)** The APLA2R in non-CR group and CR group. **(C)** *IGHV3-38* gene has a negative correlation with APLA2R. **(D–G)** CDR-H3 length, the Kyte–Doolittle index of hydrophobicity, SHM rate and germline index of IGH repertoire in CR and non-CR patients.

According to KDIGO, we divided the patients into two groups: CR and non-CR, after 6 months of therapy. We tried to investigate the diversity, hydrophobicity, GI, *IGHJ* gene, and *IGHC* gene between the two groups and found that no significant difference was observed (**Figures 6E, G**). However, we identified three *IGHV* genes (*IGHV4.61*, *IGHV4.59*, *V4 family*) were significantly increased in the CR group (**Figure 6A**). Interestingly, two of these genes (red in **Figure 6A**) in MN patients were significantly higher than those in HC (**Figure 3A**). Moreover, 11 *IGHV* and *IGHD* genes (*IGHV1.12*, *IGHV1.2*, *IGHV3.21*, *IGHV3.48*, *IGHV3.69.1*, *IGHV3.2*, *IGHV3.35*, *IGHV3.64*, *IGHV3.16*, *IGHD1.26*, *IGHD1.14*) were significantly lower in the CR group (**Figure 6A**), and six of these genes (blue in **Figure 6A**) in MN patients were significantly lower than those in HC (**Figure 3A**). In addition, APLA2R, which is the most commonly used biomarker, was found to be correlated with the CR group and non-CR group. APLA2R in the non-CR group was significantly higher than that in the CR group (**Figure 6B**). More importantly, we found the *IGHV3-38* gene had a negative correlation with APLA2R (**Figure 6C**). In addition, we found that CDR-H3 length distribution of all isotypes (P = 0.014; **Figure 6D**) and SHM rate of *IGHM* (P = 0.032; **Figure 6F**) were more prominent in the CR group.

## Discussion

B cells and T cells serve important roles in the human immune system, and understanding their receptor characteristics in human autoimmune diseases may help the development of specific immunotherapeutic interventions for diseases in the clinic (28). Each B cell expresses a single BCR (29), and the diverse range of BCRs expressed by the total B-cell population of an individual is termed the ‘BCR repertoire’. Further diversification of BCRs occurs in specialized germinal centers, in which SHM of genes that encode the variable (V) regions of antibodies may enhance BCR affinity and specificity (30). This diversification of B-cell clones after exposure to antigen is tempered by tolerance checkpoints to reduce the risk of autoimmunity (31). The peripheral BCR repertoire is thus a composite of both the naive repertoire and that generated by antigenic encounter. Abnormal use of the *IGHV* genes can exhibit abnormal isotype-specific clonal expansion or diversity in some immune-mediated diseases like SLE, Crohn’s disease, and EGPA (32). Recently, Meng Yuan et al. analyzed 294 anti-SARS-CoV-2 antibodies and found that *IGHV3-53* is the most frequently used *IGHV* gene for targeting the receptor-binding domain of the spike protein (33). Most cases of MN have circulating IgG4 autoantibody on the podocyte membrane antigen PLA2R (70%), which is characterized by glomerular subepithelial deposits of immune complexes, severe basement membrane thickening, and little or no inflammatory infiltrate (PMN); B-cell dysfunction plays a role in the pathogenesis of MN, and differential skewing of *IGHV* genes could reflect the B-cell immune status in MN patients.

We previously demonstrated the usage patterns of the TCR*β* V gene and J gene were skewed in autoimmune diseases and tumors (34–37). In our previously reported study, we analyzed the TCR*β* repertoire of the circulating T lymphocytes of MN patients (pre-therapy and post-therapy) and HC using TCR-HTS, and we found that the diversities of VJ cassette combination in the peripheral blood of MN patients were lower than those of HC. We also found the TCR*β* repertoire similarity between pre- and post-therapy could reflect the clinical outcome, and two V*β* genes in pre-therapy also had the potential to predict the therapeutic effect (38). In this work, we analyzed the IGH repertoire of PBMCs of MN patients pre- and post-therapy, as well as the PBMCs of HC, to investigate the B-cell immunity in MN patients. The patient cohort in this study included the patients from the previous reported study (38), and new patients were recruited. Changes of IGH genes in MN patients might reveal the occurrence and development of MN disease and could be used for diagnosis.

Numerous studies focused on IGH CDR-H3 clones of IgA nephropathy, with few studies about *IGHV* genes in MN. MN is an immune-related disease. If the treatment is effective, the immune status of the human body will be different from that before treatment, and the IGH repertoire will also be changed significantly, which has been reported in numerous studies (39–41). In this study, we found the frequency of *IGHV3-66* was significantly lower in the post-therapy patients than in the pre-therapy patients, and some *IGHV* genes skewed in MN patients were also significantly different between the CR and non-CR groups. All the results demonstrated that the *IGHV* gene has the potential to assist diagnosis and predict efficacy. However, due to the limited number of samples in our work, we could not confirm the performance of *IGHV* genes associated with MN disease. In fact, those biomarkers that show some potential for prognosis prediction of MN need more studies to demonstrate their accuracy and reproducibility.

The length and amino acid composition of CDR-H3 region affect recognition of antigens. Progressive reduction of CDR-H3 length and increase of highly hydrophobic and hydrophilic sequences during differentiation from immature to naive and memory B cells are paralleled by a progressive decrease in the proportion of self-reactive B-cell specificities during B-cell ontogeny (42, 43). The increased SHM indicates an enhanced *in-vivo* activation of B cells, thus suggesting abnormal activation of B cell in MN patients. In this study, these parameters were significantly different between MN and HC, CR and non-CR.

As previously discussed, proteinuria and serum creatinine may not accurately reflect disease activity and do not discriminate between immunologically active disease and irreversible structural glomerular damage (44). In fact, some studies have reported a new individualized serology-based approach that complements and refines the traditional proteinuria-driven approach, which has recently been proposed (45) and has been the subject of recent reviews (46). The hope is that we can find a personalized approach that can improve prognostic accuracy and that can provide individualized treatment for patients with MN while limiting unnecessary exposure to immunosuppressive therapy, which has many adverse effects, such as liver function damage, infection, and myelosuppression. This study indicated that some parameters of IGH repertoire could be a predictive biomarker for the treatment efficacy of MN.

## Data Availability Statement

The datasets presented in this study can be found in online repositories. The names of the repository/repositories and accession number(s) can be found in the article/**Supplementary Material**.

## Ethics Statement

The studies involving human participants were reviewed and approved by the ethics committee of the Affiliated Foshan Hospital of Sun Yat-Sen University. The patients/participants provided their written informed consent to participate in this study. Written informed consent was obtained from the individual(s) for the publication of any potentially identifiable images or data included in this article.

## Author Contributions

YZ, HL, CX, XL, PY, and YK provided patients’ samples and clinical information. ZS, ZG, XC, CZ, PL, and LZ performed the experiments. YJ performed the statistical analysis. YJ and WL analyzed and interpreted the data. YK and WL designed and supervised the study. ZS and YJ wrote the manuscript. WL did an extensive revision. All authors contributed to the article and approved the submitted version.

## Funding

This work was funded by grants from the National Natural Science Foundation of China (81972335), Foundation and Applied Basic Research Fund of Guangdong Province (2019A1515110676), Science and Technology Innovation Platform in Foshan City (FS0AA-KJ218-1301-0007), and Foshan city climbing peak plan (2019A004, 2019A025), Medical Engineering Technology Research and Development Center of Immune Repertoire in Foshan and Medical Scientific Research Foundation of Guangdong Province of China (A2021493).

## Conflict of Interest

The authors declare that the research was conducted in the absence of any commercial or financial relationships that could be construed as a potential conflict of interest.
